# A set of pedagogical recommendations for improving the integrated approach to childhood overweight and obesity: A Delphi study

**DOI:** 10.1371/journal.pone.0231245

**Published:** 2020-04-27

**Authors:** Emilie L. M. Ruiter, Gerard R. M. Molleman, Gerdine A. J. Fransen, Marlijn Wagenaar, Koos van der Velden, Rutger C. M. E. Engels

**Affiliations:** 1 Department of Primary and Community Care, Academic Collaborative Center AMPHI, Integrated Health Policy, Radboud University Medical Center, Nijmegen, the Netherlands; 2 Erasmus University Rotterdam, Rotterdam, the Netherlands; University of the Witwatersrand, SOUTH AFRICA

## Abstract

**Background:**

Tackling the increasing global problem of childhood overweight and obesity requires an integrated approach. Studies increasingly emphasize the importance of the parents’ role in interventions designed to prevent overweight in children. The aim of this study was to develop a unified set of recommendations for healthy parenting practices that can be applied by all professionals who work with children age 4–13 years and can contribute to strengthening the integrated approach to childhood overweight.

**Methods:**

A modified Delphi procedure was used to reach consensus regarding what these pedagogical recommendations should encompass. The 30 panelists were professionals and researchers who work with children age 4–13 in the domains of health care, overweight, parenting, education, nutrition, and/or sports. The procedure consisted of: *i*) extracting existing pedagogical recommendations from national guidelines and professional protocols, *ii*) appraising and prioritizing these recommendations in terms of relevance through two rounds of questionnaires, and *iii*) meeting to discuss and approve the set of recommendations.

**Results:**

Consensus was reached for one set of eleven pedagogical theme-based recommendations designed to support and instruct parents how to stimulate healthy energy balance‒related behaviors in their child. Each recommendation contained information regarding: *i*) *which* behaviors in the child and/or parent are important, *ii*) *why* this is important, and *iii*) *how* parents can stimulate this behavior by applying parenting skills in daily life. The eleven themes were: modeling, positive parenting, breakfast, varied diet, sugar-sweetened beverages, snacks, physical activity, playing sports, quantity of screen time, screen time during meals, and sleep.

**Conclusion:**

We developed a set of recommendations for healthy parenting that can be used by various professionals working with children age 4–13 and can contribute to creating an integrated approach to childhood overweight. We also developed a web-based app called “Recommendations for Healthy Parenting” as a convenient tool for following these recommendations.

## Introduction

Globally, childhood overweight and obesity are an increasing major public health problem [1, 2]. Tackling this problem requires an integrated approach that matches each local context and situation [3–8]. In the Netherlands, municipalities are increasingly encouraged to pursue an integrated health policy [9].

Parents also play an important role in facilitating, supporting, and serving as a suitable role model for their child’s healthy—and unhealthy—energy balance-related behaviors (EBRBs), as well as deal with numerous environmental factors that contribute to overweight and obesity [10, 11]. Review studies increasingly emphasize the role of parenting in the development of childhood overweight and obesity [12–15]. Greater parental involvement and holding parents responsible for implementing changes in their child’s EBRBs are generally effective for preventing and treating obesity and overweight in children [16–18]. In addition, to promote interventions that promote healthy EBRBs in overweight children, parenting styles and practices are key components and can increase the efficacy of these interventions [12, 13, 17]. To ensure that their child is within the healthy weight category, parents should use both general parenting skills and specific parenting skills regarding healthy EBRBs. In addition, parents should also be educated with respect to which EBRBs are healthiest for their child [17]. In other words, an adequate intervention should provide information regarding *what children need* (which of the child and/or parents behavior(s) is important) combined with *how to offer this* as a parent (using which general and specific EBRB-based parenting skills) [17].

However, to date, most Dutch and international obesity prevention programs have paid limited attention to parenting aspects [3, 17, 19–24]. Moreover, most programs lack a consistent and common pedagogical message for local professionals. Indeed, various professionals who work with parents and/or children generally give parents different types of pedagogical recommendations with respect to preventing childhood overweight [25]; in addition to causing confusion among parents, this nonunified approach does not contribute to establishing an integrated approach to childhood overweight.

Dutch national guidelines [26, 27], guidelines and protocols for health professionals [28–32] implemented between 2010 and 2012, and published studies [33] include a wide variety of pedagogical recommendations and describe the healthy behaviors that parents should encourage in their children (for example, drinking water instead of sugar-sweetened beverages) to prevent overweight and obesity. However, these guidelines (all developed by using national and international literature and guidelines) often lack information regarding *how* parents can use their parenting skills to stimulate these healthy behaviors in daily practice. Importantly, published studies and guidelines for professionals [26–32, 34, 35] do not provide clarity with respect to the pedagogical recommendations that professionals should provide to parents regarding how to stimulate healthy ERBRs in their child.

Therefore, the aim of this study was to develop a unified set of pedagogical recommendations that fit in well with the current national guidelines and that can be used by various professionals who work with children 4–13 years of age and can be easily incorporated into existing interventions for preventing overweight in children, thereby strengthen the integrated approach to childhood overweight. These professionals included youth healthcare (YHC) professionals, general practitioners, pediatricians, primary school teachers, school health counselors, employees at after-school care centers, pedagogues/counselors, psychologists, sports teachers, physiotherapists, and dieticians. We selected the 4–13 year age group, because many children on the primary schools had unhealthy energy balance-related behaviors. Based on data obtained from a Community Health Service in the Netherlands, around 30% of children 4–13 years of age meet the daily standards for eating fruits and vegetables, minimizing sugar-sweetened beverages, playing outside or minimizing sedentary behavior [36]. Our goal was a common set of pedagogical recommendations in order to support parents in stimulating healthy EBRBs in their 4-13-year-old child. The contents of this set of recommendations was designed to provide information regarding: *i*) *which* of the child and/or parent’s behaviors are important, and *ii*) *how* parents can stimulate this behavior by applying parenting skills and practices in daily life.

This study was based on the hypothesis that if all professionals who work with children 4–13 years of age can provide parents with a common set of fundamental pedagogical recommendations, the recommendations themselves will be more robust and may therefore have an amplifying effect, leading to reduced overweight and obesity among children [17].

## Material and methods

### Study design

We used a modified Delphi procedure to reach consensus regarding the content of a single set of recommendations for healthy parenting in order to prevent overweight in children 4–13 years of age. Our modified Delphi method consisted of two rounds of email questionnaires and a final face-to-face meeting [37, 38]. The final face-to-face meeting (third round) was not a component of the original Delphi method developed by Dalkey and Helmer [38] in 1963; rather, it was adopted from the modified Ebel procedure [39–41] and is also known as the Estimate-Talk-Estimate process [42]. The modified Delphi method was chosen because it allowed for expert interaction in the final round. This allowed members of the panel to provide further clarification on some matters and present arguments in order to justify their viewpoints. Studies have demonstrated that the modified Delphi method can be superior to the original Delphi method and perceived as highly cooperative and effective [42, 43]. During the third round anonymity was not retained. Panelists were encouraged to discuss the remaining recommendations until agreement was fully reached. This procedure is a valuable tool for reaching consensus in the absence of an existing set of recommendations [44].

All possible pedagogical recommendations, messages, advice, etc. given to parents with respect to preventing childhood overweight and/or obesity were extracted by two researchers (authors E.R. and M.W.) independently from national standards and the guidelines of the Dutch General Practitioners Association, YHC professionals, the Netherlands Nutrition Centre, and the Netherlands Sports Knowledge Centre [26, 28–32]; Thereafter the extracted list were discussed, however no different were found and the list was presented to the 2 research supervisors (authors G.M. and G.F.); these recommendations were then presented to a multidisciplinary panel of experts. The panel then reached consensus on a single set of recommendations during two rounds of questionnaires and one face-to face meeting.

The Medical Review Ethics Committee of the Region Arnhem-Nijmegen, the Netherlands approved this study (Reg. nr.: 2015–1800).

### Participants and recruitment

To ensure a sufficiently heterogeneous group of experts from practice, research, and policy, and to increase the reliability of our study [45], in March 2013 we invited a representative sample of experts to serve as panelists in our study using a purposeful sampling strategy. We included the following: *i*) professionals who work with children age 4–13 year and their parents in the following domains: healthcare, overweight, parenting, education, and sports (YHC doctors, YHC nurses, YHC policy-maker, general practitioners, pediatricians, primary school teachers, school health counselor, employee at an after-school care center, pedagogue/counselors, psychologist, sports teachers, pediatric physiotherapists, pediatric dieticians); *ii*) professionals at national research institutes (Knowledge Center for Sport and at the Nutrition Center); and *iii*) scientists who work in the following domains: parenting, childhood overweight, nutrition, and physical activity. We selected those experts who were regarded as the most prominent professionals/supervisors in their discipline in the field of healthy energy balance-related behaviors and (prevention of) childhood overweight and leading figures in our country. We asked these experts to think about what content should be included of a set of pedagogical recommendations designed to prevent childhood overweight. The participants were chosen based on their knowledge of the relevant topic and on their willingness to participate [46] and were recruited by telephone. First, researcher E.R. explained the aim of the Delphi study and then invited the professional to participate this study. To ensure a high response rate throughout the Delphi procedure, the researcher explained the Delphi procedure and why his/her participation in this study is important, as well as why his/her participation in the entire process would be necessary. Each participant was then asked whether he/she wanted to commit to the project and provided informed consent. After this telephone call, all participants received the same information by e-mail.

Currently, there is no set standard for sample size of a participants that should be included in Delphi studies, but it is generally agreed that the more members will increase the reliability of group judgments [47]. It has been suggested that a minimum number of panelist would range from 10 to 15 panel members [48, 49]. We called 34 potential panel members assuming a 25% rejection rate for the questionnaire rounds and thereafter another 50% for the meeting round, yielding a final sample of 25 panel members for the questionnaire rounds and at least 12 panel members for the meeting round.

The participants received no financial or other compensation.

### Procedure

Our modified Delphi procedure consisted of the following steps: *i*) extraction of recommendations from national guidelines and professional protocols [26–32] in order to prepare the questionnaire, *ii*) appraisal and prioritization of the recommendations in terms of relevance by having our panel of experts complete the structured questionnaires with the aim of achieving consensus in two rounds (held in March 2013 and June 2013), and *iii*) a meeting of the experts held at the end of the procedure (in June 2013). This approach was determined to be the optimal strategy by Boulkedid et al. [50] for exchanging views, resolving uncertainties, and approving the set of recommendations. Thus, a total of three rounds were held by the panel of experts. The third round, the final meeting, was held in a conference room at our university, with author E.R. serving as the moderator. During the meeting, the moderator facilitated the discussion, asked questions, probed for more information in order to clarify the participants’ comments, and controlled the input of the participants with a more dominant personality.

### Questionnaires

Approximately two weeks after being recruited by telephone and receiving an e-mail containing information regarding the study, the panelists received the first questionnaire via e-mail. We chose not to use an internet-used questionnaire, as Leece previously reported significantly lower response rates with internet-based questionnaires compared to questionnaires sent by mail [51]. The panelists were instructed to complete the questionnaire and return it by e-mail. Nineteen days after sending the first questionnaire, we sent a reminder. In total, seven weeks were needed to follow-up with initial non-respondents and to adequately analyze the results and prepare feedback for the next round. Ten weeks after sending the first questionnaire, we sent feedback to the participants together with the second questionnaire. The nature of the feedback was a qualitative summary of the results from the first questionnaire. The panelists answered the questionnaires anonymously.

#### First round: First questionnaire

The first questionnaire contained information regarding the aim of the questionnaire, why participation in this study is important, and why participation in the entire process would be necessary. In addition, the 43 existing pedagogical recommendations were listed in order to provide background information so that the panelists would be fully aware of existing recommendations/guidelines. Finally, the five questions listed in Table 1 were asked.

**Table 1:** Questions and answer options in the first and second questionnaires.

	Question	Answer options
	**Questionnaire 1**	
1	Are you familiar with these existing recommendations?	Yes/no*
2	Please indicate the extent to which you agree that this recommendation can be effective in preventing childhood obesity.	Likert scale* (ranging from 1 to 9)
3	What recommendations from either your organization or yourself would you like to see included?	Open
4	Can you suggest any general pedagogical advice for parents in order to support them in stimulating their child to have a healthy diet and engage in physical activity in daily life situations?	Open
5	From the list of recommendations to parents for preventing childhood overweight in children 4–12 years of age, which 5 do you consider the most important?	Rating of re- commendations (1–5)
	**Questionnaire 2**	
1	The panelists believe that the recommendations listed below are neither important nor effective enough to be included in a set of pedagogical recommendations for preventing childhood obesity. Do you agree?	Likert scale* (ranging 1 to 9)
2	Below, we list the top 10 most important recommendations, followed by explanations for why each recommendation is important and how parents can implement this recommendation in daily life using parenting practices. Please indicate the extent to which you agree that each recommendation should be included in the final set of recommendations.	Likert scale* (ranging 1 to 9)
3	What pedagogical recommendation(s) would you like to see added to the list?	Open

*In these questions, participants were also able to provide open comments. This method allows the participants to explain their choices and to express their views regarding the question/recommendations, thus providing useful information for developing the next round.

#### Second round: Second questionnaire

The second questionnaire provided the results (anonymously) obtained from the first round. This second questionnaire also included a list of recommendations for which consensus was reached with respect to the recommendations deemed important in a pedagogical message to parents; this list of recommendations was grouped by topic. Finally, the three questions listed in Table 1 were asked.

#### Third round: The face-to-face consensus meeting

One week before sending the second questionnaire, all panelists were invited to the third-round meeting held at Radboud University Medical Center in Nijmegen, the Netherlands. This meeting was guided by a trained moderator (author E.R.). The moderator was female and worked as a youth health care doctor (MD) and PhD-student. She followed a certified training [52] on moderating meetings. The moderator presented the final set of recommendations based on our analyses of questionnaires 1 and 2 and then facilitated the discussion, asked questions, and probed for more information in order to clarify the panelists’ comments. The meeting lasted approximately two hours. Prior to the study, there was no existing relationship between the moderator and 18 of the panelists; a prior relationship existed with the other 12 panelists, as these panelists either participated in a nationwide consultation program regarding parenting and overweight in children or were active in the same community health service as the moderator. The aim of this meeting was to discuss the ambiguities, nuances, and/or sentence structures of each recommendation in order to reach consensus regarding the final content in the set of recommendations. Each sentence in the final set of recommendations was divided into one or more of the following four categories: *i*) Full consensus reached; *ii*) Agreement with the minor changes made to this sentence based on your comments in the second round; *iii*) Near consensus, but still need to reach consensus regarding some words used in the sentence and/ or the sequence of recommendations; and *iv*) No consensus reached regarding a minor part of the recommendation. During the third round anonymity was not retained. Panelists were encouraged to discuss mainly textual ambiguities, nuances, and/or sentence structures in the remaining set of recommendations, exchange views, and resolve uncertainties, until consensus was fully reached for the final set of pedagogical recommendations.

### Data analysis

The method used to define consensus among the panelists regarding the content of pedagogical recommendations based on an analysis of the effectiveness scores panelists gave on each of the parental recommendations to prevent childhood overweight mentioned in both questionnaires. For the purpose of this study we used the following definition of consensus [53]. If a recommendation had a median effectiveness score of ≥8 or more and >80% of the experts scored this recommendation in the top tertile, then the recommendation was marked as “accepted”. If the recommendation had a median effectiveness score of <8 and <80% scored this recommendation in the top tertile, then the recommendation was marked as “not accepted” and was subsequently excluded. If neither of these criteria was met (so, If the recommendation had a median <8 and >80% of the experts scored in the top tertile or the median was ≥8 and <80% of the experts scored in the top tertile), then the recommendation was marked as “to be discussed”.

Second, two researchers (authors E.R. and M.W.) independently assigned codes to all recommendations that were accepted or marked “to be discussed”, as well as the recommendations added by the panelists, including their comments. These codes were inductively derived and were used to better categorize and interpret the opinions and comments. The code lists were discussed, and consensus was reached by the two researchers.

Third, the codes were assigned to categories (*what* is important) and themes (*why* is this behavior important and *how* can parents stimulate this behavior by applying parenting skills and practices in daily life). Fourth, using the results of questionnaire 1, questionnaire 2 was drafted. Fifth, we selected the recommendations in questionnaire 2 that had a median score of ≤2 (*“Do you agree that these recommendations should not be included*?*”*) and excluded these recommendations from the list. Sixth, we selected the recommendations in questionnaire 2 that had a median score of ≥8. Next, we prepared a summary of the comments made by the participants with respect to which changes must still be made to the set of recommendations. Finally, these results were used to develop the set of recommendations that were shown to the panelists in the third-round meeting.

## Results

### Participants

**Fig 1:**
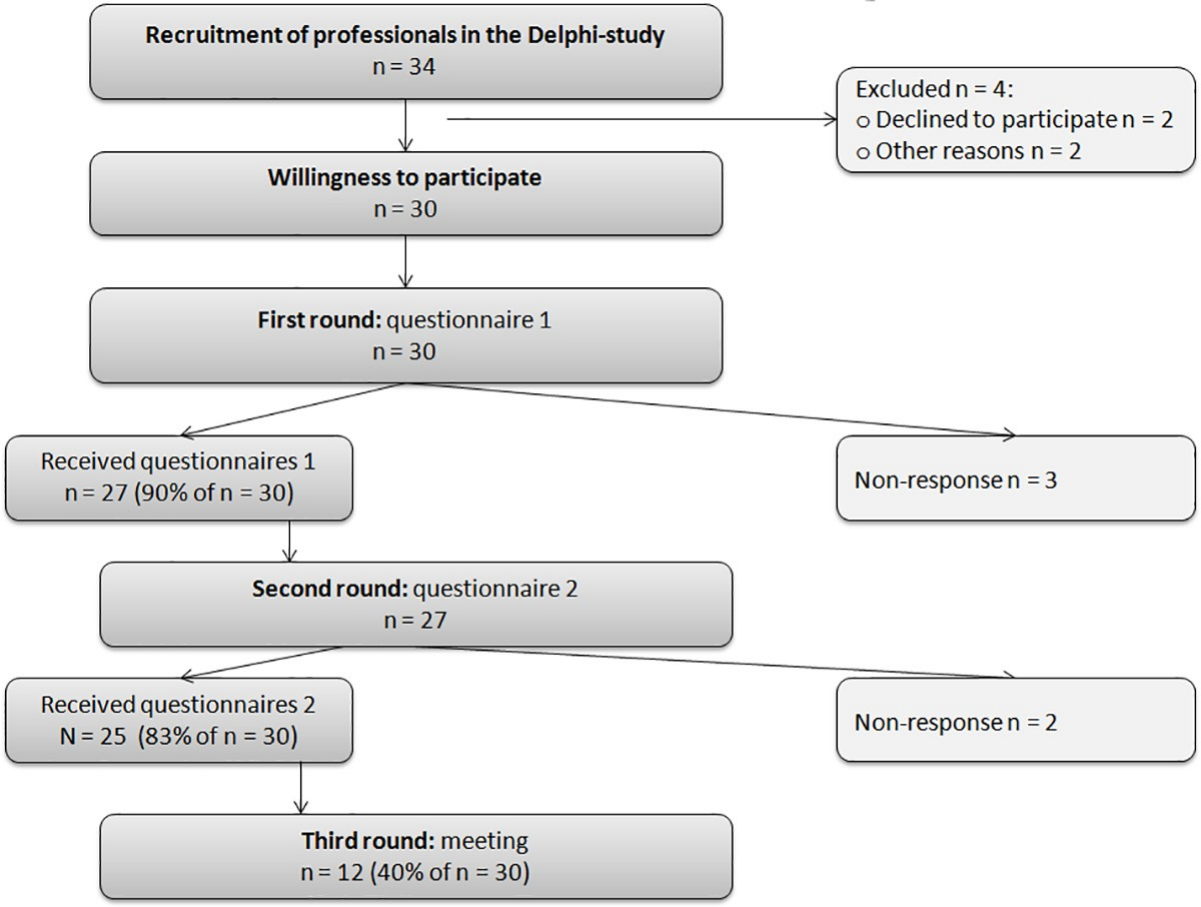
Flow-chart depicting the recruitment and participation in the three rounds of this Delphi study.

The recruitment of participants and their response rates in the three rounds of the Delphi study are summarized in Fig 1.

A total of 34 experts were invited via e-mail to participate in the Delphi study, and 30 professionals consented to participate. To ensure a representative sample of professionals from practice, research, and policy, the participants were from the following professions: 2 YHC doctors, 2 YHC nurses, 1 YHC policy-maker, 2 general practitioners, 2 pediatricians, 3 primary school teachers, 1 school health counselor, 1 employee at an after-school care center, 3 pedagogue/counselors, 1 psychologist, 2 sports teachers, 1 pediatric physiotherapists, and 2 pediatric dieticians. In addition, we invited professionals from national research institutes and scientists, specifically 1 professional working at the Knowledge Center for Sport, 1 at the Nutrition Center, and 5 scientists and university lecturers in the field of overweight and parenting. 12 Experts participated in the third round. Among these 12 experts all different occupational categories were still represented except those of a pediatrician and a primary school teacher.

### Procedure

#### First round: The first questionnaire

From the 43 recommendations presented to the panelists in questionnaire 1, 37 were accepted and therefore selected. Two additional recommendations by the panelists were also included. The codes that were assigned to these 39 recommendations were then categorized into 4 main categories (parenting, diet, physical activity, and screen time) and 39 related “why” and “how” recommendations based on *why* this recommendation is important and *how* parents can stimulate this behavior by applying parenting skills and practices in daily life. These results served as the starting point for designing the second questionnaire. These 4 main categories listed above were divided into 10 initial themes regarding *what* is important to stimulate (see Table 2).

**Table 2:** Main categories of parental recommendations.

First and second round (Main categories)	First and second round (Themes)	Third round (Themes)
1) Parenting	1) Give the right example (modeling)	1) Give the right example (modeling)
	2) Positive parenting	2) Positive parenting
2) Diet	3) Eat breakfast daily	3) Eat breakfast daily
	4) Eat a varied diet	4) Eat a varied diet
	5) Limit the consumption of sugar-sweetened beverages	5) Drink plenty of water daily
	6) Limit the number of snacks	6) Limit the number of snacks
3) Physical activity	7) ≥60 minutes of moderately intense physical activity per day	7) ≥60 minutes of moderately intense physical activity per day
	8) Play sports	8) Play sports
4) Screen time	9) ≤2 hours of screen time per day	9) ≤2 hours of screen time per day
	10) No television or computer during meals	10) No television or computer during mails
5) Sleep*	11) Adequate amount of regular sleep*	11) Adequate amount of regular sleep

*This category/theme was added after the second round

#### Second round: The second questionnaire

Consensus was reached for 5 of the 6 recommendations that were not accepted and were excluded in the first round. However, the panelists agreed that the recommendation—encouraging adequate and regular sleep—cannot be excluded; therefore, a 5th main category—sleep—was added. In addition, consensus was reached regarding the 10 themes that emerged from the first round, and 38 of the 39 “why” and “how” recommendations in questionnaire 1 were accepted; one recommendation was not accepted and therefore excluded. Thus, after the second round, consensus was reached regarding the following 5 main categories: parenting, diet, physical activity, screen time, and sleep; these categories were then divided into 11 themes (Table 2) and 39 recommendations (including a new recommendation regarding). These results served as the starting point for preparing the third round.

#### Third round: The face-to-face consensus meeting

The 39 recommendations were assigned to one of the following four levels of consensus: *i*) fully consensus (13 recommendations), *ii*) agreement with the minor change made to this recommendation based on your comments in the 2^nd^ round (5 recommendations), *iii*) almost consensus, only need to obtain consensus on some words used in the sentence or the sequence of recommendations (19 recommendations), and *iv*) No consensus regarding a small part of the recommendation (2 recommendations). In this third round, no recommendations were either removed or added, and full consensus was reached regarding all aspects in the set of recommendations. In all 11 themes, the sequence of recommendations was arranged systematically, and all recommendations were formulated using positive language. Each of these eleven themes contained information regarding: *i*) *which* of the child’s and/or parents’ behavior is important, *ii*) *why* is this important, and *iii*) *how* can parents stimulate this behavior by applying parenting skills and practices in daily life (see Table 3 for two examples). This approach resulted in consensus being reach for a single set of 11 pedagogical recommendations for supporting parents with respect to stimulating healthy EBRBs in their child.

**Table 3:** Brief examples of few pedagogical recommendations for achieving two recommendations from the third round (drink water daily and engage in ≥60 minutes of moderately intense physical activity each day).

What	Why	How
Drink water daily	Nearly all processed drinks contain a high amount of sugar, which is unhealthy and can cause overweight and cavities in children. Thus, drinking water is a healthier choice.	Limit the consumption of sugar-sweetened beverages by having as few sugar-sweetened beverages as possible in your house. Thus, your child will not be tempted and will drink water when thirsty. Set a good example by drinking sugar-free beverages or few sugar-sweetened beverages in the presence of your child. For example, water, unsweetened tea, or syrup diluted with a lot of water.
Engage in ≥60 minutes of moderately intense physical activity daily	When your child gets enough exercise, he/she feels healthier, is stronger, and performs better at school. Moreover, regular exercise reduces the risk of developing diabetes, overweight, and depression. You can easily include more exercise in your child’s daily life.	Make exercise fun by participating yourself and by inviting your child’s friends and sibling to participate. Ensure that exercise equipment such as a bicycle, soccer ball, etc. are readily available. Place computers, tablets, etc. out of sight.

### Web app

**Fig 2:**
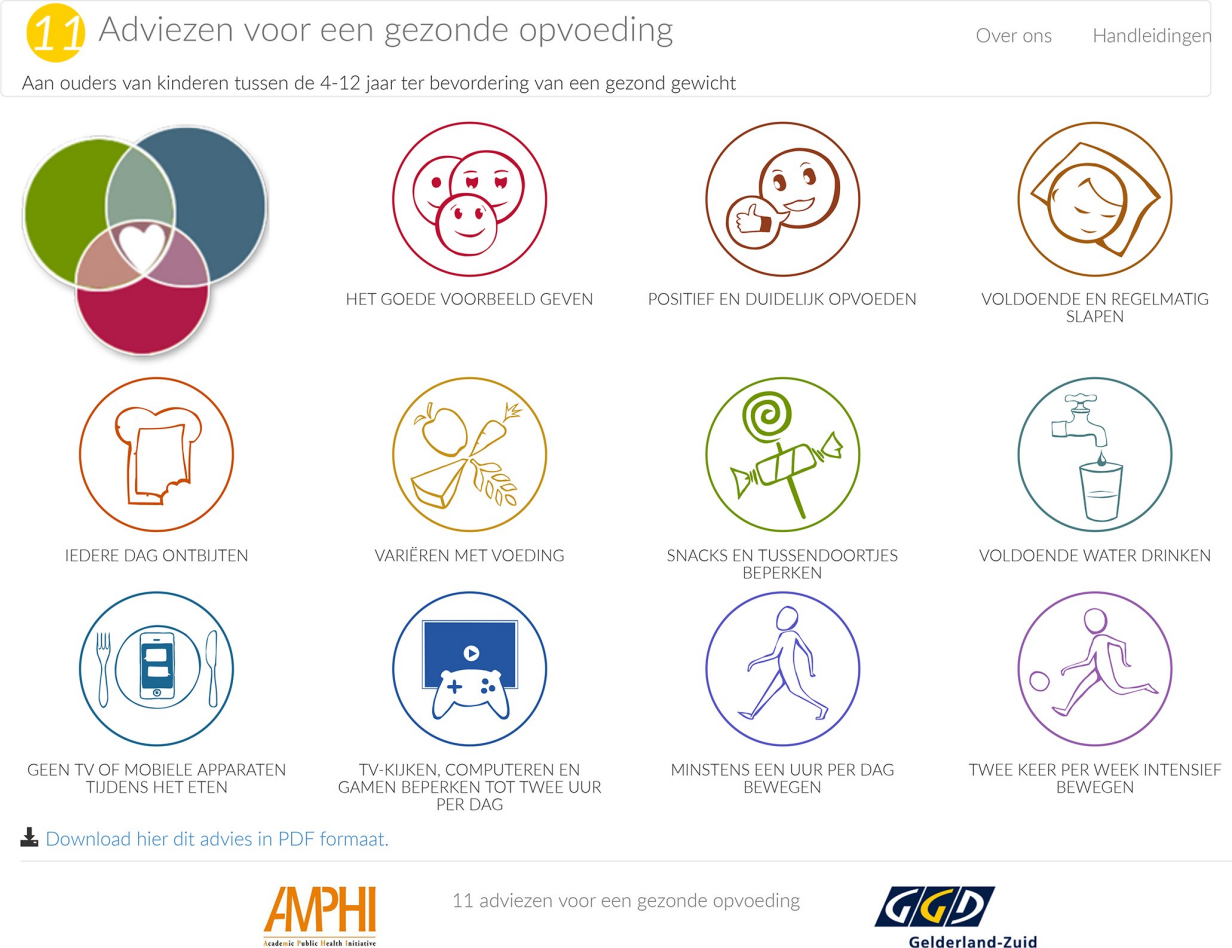
Screenshot of the web app “11 recommendations for healthy parenting”.

Next, the 11 recommendations that were obtained as a result of this Delphi study were incorporated into a simple, user-friendly web app called “11 Recommendations for Healthy Parenting”; this web app, which is currently available only in Dutch at www.adviezenvooreengezondeopvoeding.nl, is shown in Fig 2. The text used in the web app was written at the B1 level.

## Discussion

Using a modified Delphi procedure, we developed a set of 11 pedagogical recommendations that can be used by a broad range of professionals in order to help strengthen the integrated approach to reducing overweight and obesity in children, particularly children 4–13 years of age. In this procedure, a panel of experts comprised of a heterogeneous mixtures of professionals reached consensus regarding the content of this set of recommendations. The recommendations were based on existing national guidelines in the Netherlands, professional protocols, and recommendations from the panel. Each of these 11 recommendations contains information regarding: *i*) *which* of the child’s or parents’ behavior is considered important, *ii*) *why* this behavior is important, and *iii*) *how* parents can stimulate this behavior using parenting skills and parenting practices in daily life. The systematic structure of each recommendation and the explicit description of each practical recommendation represent an advance in terms of helping parents reduce the risk of overweight and obesity in their child. Importantly, this comprehensive set of recommendations is manageable, clear, unambiguous, easy to use, and supported by a broad variety of professionals. Moreover, each recommendation is formulated using positive language wherever possible. Finally, these 11 recommendations have been integrated into in a convenient, user-friendly web app entitled “11 Recommendations for Healthy Parenting”, available at www.adviezenvooreengezondeopvoeding.nl.

### Strengths and limitations

The main strength of our study is the heterogeneity of professions among the 30 participants, which covers 16 different professions, ensuring the panel was representative of all professionals who will be affected by the results. Moreover, we selected a panel with a wide range of expertise and broad knowledge regarding pedagogical recommendations and how to communicate these recommendations to the parents of school-age children, thereby contributing to the reliability of our results [45]. Studies in the field of psychology suggest that heterogeneity among a decision-making group can lead to better performance than a homogeneous group of decision-makers [54]. Thereafter, diversity among panelists can provide a variety of perspectives and yield a wide range of alternatives [47]. A second strength is the number of participants and the relatively high response rate in the first two rounds (which were anonymously), thereby resulting in a reliable set of recommendations. Currently, no clear guidelines are available with respect to the number of participants that should be include in studies applying the Delphi survey approach, as the participants are selected with a specific purpose in mind and based on the problem being investigated. Nevertheless, Fitch and colleagues suggest that 7–15 experts per panel are needed in order to develop a reliable set of indicators [48]. In addition, 2–3 rounds are recommended, which is consistent with our approach. However, strikingly little scientific evidence is available with respect to the optimal number of rounds in a Delphi study. In our study, complete consensus was reached after three rounds.

Feedback is an essential component of the Delphi procedure. Murphy et al. recommend that when selecting healthcare quality indicators, the feedback should include qualitative comments and statistical measures such as the panel results (e.g., median, lowest, and highest ratings) and the participant's response [47], and individual feedback should be reported. Nevertheless, in our study we only provided the panelists with qualitative feedback. We did not report quantitative feedback to the panel, and we did not report individual feedback, as there was a high degree of consensus even in the first round. Nevertheless, we believe the outcome would have been similar if we included quantitative and individual feedback. It is probable that the experts who were invited properly represent the experts in this field. However, another possible limitation is that the results may not necessarily represent the views of all the invited experts, given that 17% of invited participants did not respond in the first and/or second round. Nevertheless, the phone and e-mail messages that we received from the non-responders indicate that these experts likely chose not to participate due simply to a lack of time, and we found no indication of a possible selection bias. Another possible limitation is that we only used Dutch national guidelines. With this, there is a chance that we have missed international recommendations. However, our Dutch guidelines are based on extensive national and international literature, research and debate by a multidisciplinary working group composed of representatives of all relevant specialisms involved with the diagnosis and treatment of obesity. The guidelines are nationally well implemented and accepted by professionals [55]. Additionally, in the Delphi questionnaire, we asked the panelists, including researchers who are very well informed about (the recent) international literature, if they have additional recommendations. Based on this, we believe that our results, the 11 recommendations, fit in well with the current national guidelines and knowledge from international literature is included.

### Implications for practice, policy, and future research

The resulting set of 11 recommendations for healthy parenting that fit in well with the current national guidelines and can be used by a variety of professionals who work with children 4–13 years of age. We believe that if these professionals from a wide range of backgrounds provide parents with this set of fundamental pedagogical recommendations, the recommendations will be more effective and may therefore have an amplifying effect, helping strengthen the integrated approach to reducing childhood overweight. To help professionals implement this set of recommendations, we designed a web app entitled “11 Recommendations for Healthy Parenting”. Providing these recommendations via a web app is likely to help them reach a wide audience, as studies have shown that parents are less willing to physically go to an informational meeting [56–58]. Offering information via the web is a cheaper and simpler method. This web app is a valuable tool that can be used to help a heterogeneous group of professionals support parents with respect to stimulating healthy EBRBs in their child using positive parenting skills and practices, thereby strengthening the integrated approach to childhood overweight. Importantly, this web app can be used to easily convey the recommendations to parents and to promote discussions with parents. The URL for the web app can be given to parents, enabling them to read the recommendations at their leisure. The web app can also be downloaded for free to any smartphone, tablet, or computer, and it can be placed directly on the desktop or home screen. Each recommendation has also been converted into an easily recognizable icon; by selecting one of the 11 icons, the corresponding recommendations appear and can be downloaded in PDF format or printed. In addition, this web app can be added to existing interventions designed to prevent childhood overweight, thereby adding a pedagogical component.

Further research is needed in order to implement and evaluate/verify the applicability, satisfaction, and effectiveness of this web app. However, its emphasis on parental involvement and pedagogical aspects, the focus on various topics, and the fact that it can contribute to developing an integrated approach to childhood overweight are all promising features with respect to its effectiveness.

## Conclusions

The result of our Delphi study yielded a single set of clear recommendations for healthy parenting for use by various professionals who work with children 4–13 years of age, thereby helping strengthen the integrated approach to childhood overweight. Moreover, we developed and released a web app entitled “11 Recommendations for Healthy Parenting” to provide a convenient tool for easily communicating these recommendations to parents.

## Supporting information

S1 File (DOC)

S2 File (DOC)

S3 File (DOC)

S4 File (DOC)

S1 Data (XLS)

S2 Data (XLS)

## Abbreviations

EBRB: energy balance-related behavior
YHC: youth healthcare
SSBs: sugar-sweetened beverages
