# Supplementary material for: A set of pedagogical recommendations for improving the integrated approach to childhood overweight and obesity: A Delphi study
Source: PLoS One. 2020 Apr 27;15(4):e0231245. doi: 10.1371/journal.pone.0231245 (PMC7185684; doi:10.1371/journal.pone.0231245)
Supplement: S1 File — (DOC) [file pone.0231245.s001.doc]

| 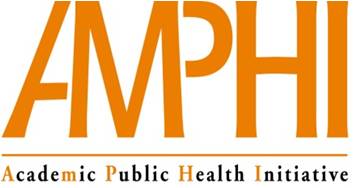 |  |
| --- | --- |

**Vragenlijst ronde 1**

**Maart 2013**

**Consensus krijgen over de inhoud van een pedagogische boodschap voor ouders ter preventie van overgewicht bij kinderen tussen de 4 en 13 jaar: een Delphi studie**

**Inleiding**

Uit de literatuur en richtlijnen betreffende (de preventie van) overgewicht bij kinderen, komen een aantal belangrijke determinanten en bijbehorende adviezen naar voren die we hieronder voor u zullen opnoemen. Zo maken de Jeugd Gezondheidszorg (JGZ), het Voedingscentrum en Nederlands Instituut voor Sport en Bewegen (NISB) gebruik van de **SO** die **(B)BOFT**-factoren. Daarnaast heeft het Nederlands Genoodschap voor Huisartsen (NHG) een aantal richtlijnen opgesteld ter preventie van overgewicht bij kinderen, de NHG-patiëntenbrief: “Overgewicht bij kinderen”. Naast de hierboven genoemde organisaties zijn er nog meer professionals, instellingen en betrokkenen die regels en richtlijnen hebben opgesteld of meningen hebben geformuleerd ten behoeve van de preventie van overgewicht bij kinderen tussen de 4 en 13 jaar oud.

| De afkorting ‘**SO** die **(B)BOFT**’ staat voor: | Bijbehorende **adviezen aan ouders** zijn**:** |
| --- | --- |
| - Stimuleren van voldoende en regelmatige **S**laap | - Minstens 10 uur slaap per nacht |
| - Stimuleren van competente **O**pvoedingsstijlen | - Duidelijke regels en afspraken met je kind en tegelijkertijd liefde en warmte geven. |
| - Bevorderen van dagelijks **B**uitenspelen en bewegen | - Ouders bewegen zelf, zodat kinderen voorbeeld hebben. - Minder autogebruik. - >1uur/dag fietsen, lopen of sporten. - Samen met kinderen zwemmen, fietsen of lopen. |
| - **O**ntbijt iedere dag | - Ontbijten bij voorkeur in gezinsverband. - Gezond ontbijt aanbieden. - Geen TV bij ontbijt. |
| - **F**ris water uit de kraan, laat **F**risdranken en andere gezoete drankjes staan | - Geen gezoete dranken kopen. - Geen of maximaal 1 glas gezoete frisdrank per dag. - Kinderen drinken water, thee of vruchtensap aangelengd met water. |
| - **T**v en pc? Zeg wat vaker nee. | - Kinderen kijken niet langer dan 2 uur per dag tv en of computeren. - Nooit langer Tv kijken of computeren dan bewegen. - Geen tv op de kinderslaapkamer. |

Ook het stimuleren van **B**orstvoeding behoort tot deze regels, maar omdat wij ons willen richten op de leeftijdsgroep tussen 4 en 13 jaar, wordt deze regel buiten beschouwing gelaten.

De determinanten in de NHG-patiëntenbrief komen grotendeels overeen met de SO die BOFT-factoren die door de JGZ, het voedingscentrum en het NISB gebruikt worden.

| De determinanten die de NHG stelt in de de **NHG-patiëntenbrief: Overgewicht bij kinderen**, zijn: | Bijbehorende **adviezen aan ouders** zijn: |
| --- | --- |
| - Meer bewegen voor een kind met overgewicht | - Maak afspraken met uw kind om te zorgen dat het niet te lang achter de computer of de tv zit. - Zorg dat uw kind regelmatig buiten speelt, boodschappen doet op de fiets of de hond uitlaat. - Een kind moet dagelijks minstens een uur actief bewegen. Forceer het niet maar doe zelf mee en maak het leuk. Vraag ook vriendjes, broertjes of zusjes om mee te doen. - Schrijf uw kind in bij een sportvereniging en ga regelmatig kijken naar trainingen of wedstrijden om het te stimuleren. - Als uw kind zwemdiploma A heeft gehaald, laat uw kind dan doorgaan voor diploma B en C. - Bedenk een activiteit die u met het hele gezin kunt doen, zoals samen een boswandeling, strandwandeling of fietstocht maken of samen zwemmen in het weekend. - Loop samen naar school, neem vaker de fiets in plaats van de tram, bus of auto. - Alle kleine beetjes extra lichaamsbeweging helpen. De voedingsmiddelen die uw kind binnenkrijgt worden dan beter verbruikt. Uw kind krijgt steeds meer spieren en minder vet. Het raakt minder snel buiten adem en voelt zich steeds fitter. |
| - Gezond eten voor een kind met overgewicht | Om te zorgen dat uw kind niet te zwaar wordt, is het nodig dat het anders gaat eten.   - Zorg voor afwisseling in het eten. In groente, fruit en volkoren producten zitten veel waardevolle voedingsstoffen en weinig calorieën. Ze geven een vol gevoel, waardoor uw kind minder snel te veel eet. - Kies voor mager vlees en voor magere of halfvolle melkproducten. - Gebruik weinig vet bij het koken. - Ongeveer een liter drinken per dag is belangrijk: naast melk vooral ook water of thee zonder suiker, en soms ook vers sap. - Door gezond en gevariëerd te eten krijgt uw kind alle voedingstoffen binnen die het nodig heeft. - Vitaminepillen zijn niet nodig. - Op de website van [het Voedingscentrum](http://www.voedingscentrum.nl/) kunt u de leeftijd en het geslacht van uw kind intypen. Dan krijgt u een overzicht van wat uw kind per dag het beste kan eten. - Eet op vaste tijden. - Sla het ontbijt niet over. - Drie maaltijden per dag is prima. - Geef hooguit vier keer per dag een gezond tussendoortje, bijvoorbeeld: fruit, een volkorenbiscuit, soepstengel of rijstwafel, een doosje rozijnen, stukjes komkommer of wortel. Geef zo min mogelijk frisdrank, snoep of chips. - Laat uw kind alleen bij uitzondering snoepen op feestjes of in het weekend en ook dan alleen weinig. - Geef zelf het goede voorbeeld. Doe mee aan de regelmaat en aan de gezonde voeding. - Gebruik eten niet als zoethoudertje om uw kind af te leiden, te troosten of te belonen. - Geef uw kind aandacht, luister naar wat het te zeggen heeft, geef het een extra knuffel of lees samen een boekje. Als een kind de juiste aandacht krijgt, heeft het minder behoefte om te snoepen. |

**Vragen:**

*Eerst vragen wij u iets over uw bekendheid met en uw mening over de inhoud van bovenstaande SO die BOFT-factoren en de NHG-patiëntenbrief: “Overgewicht bij kinderen”.*

1. Bent u bekend met de SO die BOFT-factoren? Kies ja of nee. Daarnaast kunt u in het opmerkingenveld de keuze voor uw antwoord indien nodig toelichten.

| **Antwoord** | **Opmerkingen** |
| --- | --- |
| Ja/nee  *(Verwijder het antwoord wat niet van toepassing is).* | *Vul hier uw eventuele opmerkingen in.* |

1. Kunt u per SO die BOFT-factor aangeven in hoeverre u het eens bent met de effectiviteit van deze factor ter preventie van overgewicht bij kinderen? Dit kunt u aangeven op een **schaal van 1 tot 9**. Hierbij betekent **1** dat u het **helemaal oneens** bent en **9** dat u het **helemaal eens** bent met de effectiviteit van deze factor. Daarnaast kunt u in het opmerkingenveld de keuze voor uw antwoord toelichten.

| **SO die BOFT-factoren** | **Deze factor ter preventie van overgewicht bij kinderen 4-13 jr is zeer effectief.**  **Noteer hieronder een cijfer tussen de 1-9 .** | **Opmerkingen: licht uw antwoord toe.** |
| --- | --- | --- |
| Stimuleren van voldoende en regelmatige slaap |  |  |
| Stimuleren van competente opvoedingsstijlen |  |  |
| Bevorderen van dagelijks buitenspelen en bewegen |  |  |
| Ontbijt iedere dag |  |  |
| Beperken van frisdranken en andere gezoete drankjes |  |  |
| Tv en pc? Zeg wat vaker nee |  |  |

1. Bent u bekend met de NHG-patiëntenbrief: “Overgewicht bij kinderen”? Kies ja of nee. Daarnaast kunt u in het opmerkingenveld de keuze voor uw antwoord indien nodig toelichten.

| **Antwoord** | **Opmerkingen** |
| --- | --- |
| Ja/nee  *(Verwijder het antwoord wat niet van toepassing is).* | *Vul hier uw eventuele opmerkingen in.* |

1. Kunt u voor de adviezen aan ouders behorende bij de ‘**SO die BOFT-factoren**’ en de ‘**NHG-patiëntenbrief: “Overgewicht bij kinderen**” aangeven in hoeverre u deze adviezen effectief vindt ter preventie van overgewicht bij kinderen? Dit kunt u aangeven op een **schaal van 1 tot 9**. Hierbij betekent **1** dat u het **helemaal oneens** bent en **9** dat u het **helemaal eens** bent met de effectiviteit van het advies. Daarnaast kunt u in het opmerkingenveld de keuze voor uw antwoord toelichten.

| **Adviezen aan ouders** | **Dit advies ter preventie van overgewicht bij kinderen 4-13 jr is zeer effectief.**  **Noteer hieronder een cijfer tussen de 1-9 .** | **Opmerkingen: licht uw antwoord toe.** |
| --- | --- | --- |
| Minstens 10 uur slaap per nacht |  |  |
| Duidelijke regels en afspraken met je kind en tegelijkertijd liefde en warmte geven. |  |  |
| Ouders bewegen zelf, zodat kinderen voorbeeld hebben. |  |  |
| Minder autogebruik. |  |  |
| >1uur/dag fietsen, lopen of sporten. |  |  |
| Samen met kinderen zwemmen, fietsen of lopen. |  |  |
| Ontbijten bij voorkeur in gezinsverband. |  |  |
| Gezond ontbijt aanbieden. |  |  |
| Geen TV bij ontbijt. |  |  |
| Geen gezoete dranken kopen. |  |  |
| Geen of maximaal 1 glas gezoete frisdrank per dag. |  |  |
| Kinderen drinken water, thee of vruchtensap aangelengd met water. |  |  |
| Kinderen kijken niet langer dan 2 uur per dag tv en of computeren. |  |  |
| Nooit langer tv kijken of computeren dan bewegen per dag. |  |  |
| Geen tv op de kinderslaapkamer. |  |  |
| Maak afspraken met uw kind om te zorgen dat het niet te lang achter de computer of de tv zit. |  |  |
| Zorg dat uw kind regelmatig buiten speelt, boodschappen doet op de fiets of de hond uitlaat. |  |  |
| Een kind moet dagelijks minstens een uur actief bewegen. Forceer het niet maar doe zelf mee en maak het leuk. Vraag ook vriendjes, broertjes of zusjes om mee te doen. |  |  |
| Schrijf uw kind in bij een sportvereniging en ga regelmatig kijken naar trainingen of wedstrijden om het te stimuleren. |  |  |
| Als uw kind zwemdiploma A heeft gehaald, laat uw kind dan doorgaan voor diploma B en C. |  |  |
| Bedenk een activiteit die u met het hele gezin kunt doen, zoals samen een boswandeling, strandwandeling of fietstocht maken of samen zwemmen in het weekend. |  |  |
| Loop samen naar school, neem vaker de fiets in plaats van de tram, bus of auto. |  |  |
| Alle kleine beetjes extra lichaamsbeweging helpen. De voedingsmiddelen die uw kind binnenkrijgt worden dan beter verbruikt. Uw kind krijgt steeds meer spieren en minder vet. Het raakt minder snel buiten adem en voelt zich steeds fitter. |  |  |
| Zorg voor afwisseling in het eten. In groente, fruit en volkoren producten zitten veel waardevolle voedingsstoffen en weinig calorieën. Ze geven een vol gevoel, waardoor uw kind minder snel te veel eet. |  |  |
| Kies voor mager vlees en voor magere of halfvolle melkproducten. |  |  |
| Gebruik weinig vet bij het koken. |  |  |
| Ongeveer een liter drinken per dag is belangrijk: naast melk vooral ook water of thee zonder suiker, en soms ook vers sap. |  |  |
| Door gezond en gevarieerd te eten krijgt uw kind alle voedingstoffen binnen die het nodig heeft. |  |  |
| Vitaminepillen zijn niet nodig. |  |  |
| Op de website van [het Voedingscentrum](http://www.voedingscentrum.nl/) kunt u de leeftijd en het geslacht van uw kind intypen. Dan krijgt u een overzicht van wat uw kind per dag het beste kan eten. |  |  |
| Eet op vaste tijden. |  |  |
| Sla het ontbijt niet over. |  |  |
| Drie maaltijden per dag is prima. Geef hooguit vier keer per dag een gezond tussendoortje, bijvoorbeeld: fruit, een volkerenbiscuit, soepstengel of rijstwafel, een doosje rozijnen, stukjes komkommer of wortel. Geef zo min mogelijk frisdrank, snoep of chips. |  |  |
| Laat uw kind alleen bij uitzondering snoepen op feestjes of in het weekend en ook dan alleen weinig. |  |  |
| Geef zelf het goede voorbeeld. Doe mee aan de regelmaat en aan de gezonde voeding. |  |  |
| Gebruik eten niet als zoethoudertje om uw kind af te leiden, te troosten of te belonen. |  |  |
| Geef uw kind aandacht, luister naar wat het te zeggen heeft, geef het een extra knuffel of lees samen een boekje. Als een kind de juiste aandacht krijgt, heeft het minder behoefte om te snoepen. |  |  |

1. Zoals in de inleiding al genoemd, kunt u als expert ook in uw eigen beroepsgroep adviezen en richtlijnen hebben, dan wel uw eigen ideeën hebben over de pedagogische boodschap aan ouders om overgewicht bij kinderen tussen 4 en 13 jaar te voorkomen. Voor deze ideeën hoeft noch een wetenschappelijke onderbouwing te zijn noch ondersteuning door andere experts. Welke adviezen, van andere organisaties of uzelf, mist u nog en zou u willen toevoegen? Licht uw antwoord toe.

| **Andere adviezen of uw eigen ideeën** | **Licht uw antwoord toe** |
| --- | --- |
|  |  |
|  |  |
|  |  |
|  |  |
|  |  |
|  |  |
|  |  |
|  |  |
|  |  |

1. Als u naar bovengenoemde factoren, richtlijnen en adviezen aan ouders kijkt, zijn er dan (nog) algemene pedagogische adviezen die ouders in de dagelijkse situatie helpen hun kind gezonder te laten eten en voldoende te bewegen? Het gaat hierbij dus om de algemene opvoeding (general parenting): de mate waarin ouders duidelijke afspraken maken met hun kind maken, regels stellen, steunen, belonen en straffen.

| **Andere algemene opvoedingsregels** | **Licht uw antwoord toe** |
| --- | --- |
|  |  |
|  |  |
|  |  |
|  |  |
|  |  |
|  |  |
|  |  |
|  |  |
|  |  |
|  |  |
|  |  |
|  |  |
|  |  |

1. Kunt u een top 5 maken van de adviezen/boodschappen aan ouders ter preventie van overgewicht bij kinderen tussen de 4 en 13 jaar, die volgens u het meest belangrijk zijn? Begin met het advies/de boodschap die u het meest belangrijk vindt.

| **Top 5 van adviezen/boodschappen aan ouders ter preventie van overgewicht bij kinderen tussen den 4 en 13 jaar** | **Licht uw antwoord toe** |
| --- | --- |
| 1. |  |
| 2. |  |
| 3. |  |
| 4. |  |
| 5. |  |

1. Kent u nog experts die wij zouden kunnen benaderen voor deze of de volgende ronde van deze Delphi studie? Zou u de naam, functie, e-mailadres en eventueel telefoonnummer van deze persoon willen noteren, zodat wij de desbetreffende persoon kunnen uitnodigen voor deelname aan ons onderzoek.

Volledige naam:

Functie/beroep:

E-mailadres:

Telefoonnummer:

**Dit is het einde van de 1^e^ vragenlijst.**

**Hartelijk bedankt voor het invullen van deze vragenlijst. Zou u de ingevulde vragenlijst vóór *<datum>* naar ons willen retourneren via:**

**Mocht u vragen en/of opmerkingen hebben dan kunt u deze hieronder kwijt.**

|  |
| --- |

Voor vragen over de vragenlijst en/of het invullen daarvan kunt u contact opnemen met:
