# Supplementary material for: A set of pedagogical recommendations for improving the integrated approach to childhood overweight and obesity: A Delphi study
Source: PLoS One. 2020 Apr 27;15(4):e0231245. doi: 10.1371/journal.pone.0231245 (PMC7185684; doi:10.1371/journal.pone.0231245)
Supplement: S2 File — (DOC) [file pone.0231245.s002.doc]

| 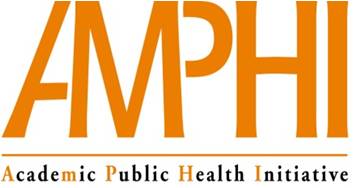 |  |
| --- | --- |

**Vragenlijst ronde 2**

**Juni 2013**

**Consensus krijgen over de inhoud van een pedagogische boodschap voor ouders ter preventie van overgewicht bij kinderen tussen de 4 en 13 jaar: een Delphi studie**

**Inleiding**

Uit de resultaten van de eerste ronde van onze Delphi studie blijkt dat er onder de verschillende deelnemende professionals aan onze Delphi studie al veel consensus bestaat. Hieronder vindt u de samengevoegde maatregelen waarover u allen het eens bent dat deze belangrijk zijn in een pedagogische boodschap naar ouders:

1. Opvoeding
   1. Stimuleren van een competente opvoedingsstijl, ofwel een authoritatieve opvoedingsstijl. *Wat u allen vindt passen onder een competente opvoedingsstijl is:*
      1. Structuur (binnen het huishouden en gezin);
      2. Duidelijke regels en afspraken en consequent zijn;
      3. Liefde, warmte en aandacht geven aan je kind, samen leuke dingen doen;
      4. Luisteren naar je kind;
      5. Het kind belonen of complimenten geven wanneer hij/zij iets goeds doet.
   2. Het goede voorbeeld geven.
   3. Eten moet niet gebruikt worden als zoethoudertje om uw kind te troosten, af te leiden of te belonen.
   4. Leg geen nadruk op afvallen/diëten.
   5. Zorg dat ongezonde producten of activiteiten (bijvoorbeeld chips, de iPad) niet zichtbaar zijn, terwijl gezonde producten en activiteiten (bijvoorbeeld fruit, een fiets of bal) wel zichtbaar zijn.

*Gezond eten bevorderen door de punten genoemd onder opvoeding.*

1. Eten
   1. Ontbijt iedere dag, liefst samen en aan tafel.
   2. 3 hoofdmaaltijden en maximaal 3 tussendoortjes op vaste tijden, liefst samen aan tafel. Zie voor de hoeveelheden de richtlijnen van het voedingscentrum.
   3. Varieer met eten, zodat uw kind voldoende verschillende voedingsstoffen binnen krijgt. Ook is het belangrijk dat u uw kind alles laat proeven.
2. Frisdrank en snoepen/snacks
   1. Beperk de inname van zoete dranken, zoals vruchtensappen, ranja, ice-tea en frisdranken. *Er is nog geen consensus over een concreet drinkadvies. Deze kan zijn per dag: 2 glazen melk, 1 glas zoet (bijv ranja/vruchtensap/multivitamine/ frisdrank) overig water, thee zonder suiker. Liefst vruchtensappen mengen met water.*
   2. Beperken snoep/snacks. *Er bestaat nog geen consensus over hoe vaak snoepen/snacks.*

*Voldoende bewegen en verminderen van inactiviteit bevorderen door de punten genoemd onder opvoeding.*

1. Bewegen
   1. 60 minuten minimaal bewegen per dag: zoals (naar school) lopen of fietsen, rennen, zwemmen, hond uitlaten.
   2. Samen met het gezin of met vriendjes bewegen, zodat het leuk blijft.
   3. Beslis samen met uw kind welke vorm van bewegen hij/zij leuk vindt.
   4. Vraag financiële hulp bij gemeente voor bijvoorbeeld sportsubsidie, wanneer u dit nodig heeft.
2. Tv en pc
   1. Niet langer dan 2 uur per dag tv kijken en computeren. Dit geldt ook voor apparaten als de Xbox en de iPad.
   2. Geen tv of computer op de kinderkamer.

Daarnaast had u ook aanmerkingen en/of aanvullingen op de adviezen aan ouders uit de ‘SO die BOFT-factoren’ en de richtlijnen uit de ‘NHG-patiëntenbrief’. Er waren echter ook adviezen die u allen minder belangrijk vond.

Op basis van bovenstaande bevindingen is deze tweede vragenlijst opgesteld.

**Alvast hartelijk bedankt voor uw antwoorden!**

**Vragen:**

1. De volgende adviezen werden door de meeste deelnemende professionals als NIET belangrijk of NIET effectief genoeg beschouwd om op te nemen in een pedagogische boodschap ter preventie van overgewicht bij kinderen. Kunt u per advies aangeven in hoeverre u het eens bent dat dit advies **NIET** door ons wordt opgenomen in de pedagogische boodschap. Dit kunt u aangeven op een **schaal van 1 tot 9**. Hierbij betekent **1** dat u het **helemaal oneens** bent en **9** dat u het **helemaal eens** bent met het NIET opnemen van dit advies. Daarnaast kunt u in het opmerkingenveld de keuze voor uw antwoord toelichten.

| **Adviezen aan ouders** | **Dit advies ter preventie van overgewicht bij kinderen 4-13 moet NIET opgenomen worden in de pedagogische boodschap.**  **Noteer hieronder een cijfer tussen de 1-9 .** | **Opmerkingen: licht uw antwoord toe.** |
| --- | --- | --- |
| Stimuleren van voldoende en regelmatige slaap |  |  |
| Minstens 10 uur slaap per nacht |  |  |
| Schrijf uw kind in bij een sportvereniging en ga regelmatig kijken naar trainingen of wedstrijden om het te stimuleren |  |  |
| Als uw kind zwemdiploma A heeft gehaald, laat uw kind dan doorgaan voor diploma B en C |  |  |
| Vitaminepillen zijn niet nodig |  |  |
| Laat uw kind alleen bij uitzondering snoepen of feestjes of in het weekend en ook dan alleen weinig |  |  |

1. *Hieronder volgt een top 10 van mogelijke pedagogische boodschappen die professionals kunnen uitdragen naar ouders, zodat zij weten hoe zij overgewicht bij kinderen tussen de 4 en 13 jaar kunnen voorkomen. Deze boodschappen zijn opgebouwd uit de adviezen waarover consensus is bereikt. De boodschappen zijn onderverdeeld in een ‘****WAT****’-boodschap en een ‘****HOE****’-boodschap. De ‘****WAT****’-boodschap bestaat uit concrete boodschappen, welke zijn samengesteld op basis van samengevoegde adviezen waarover consensus is bereikt. In de ‘****HOE****’-boodschap zijn pedagogische tips, gebaseerd op de adviezen waarover door u consensus is bereikt, gegeven om de ‘****WAT****’-boodschap toe te lichten aan ouders.*

Kunt u per ‘**HOE**’-boodschap aangeven in hoeverre u het eens bent dat dit advies door ons wordt opgenomen in de pedagogische boodschap. Dit kunt u aangeven op een **schaal van 1 tot 9**. Hierbij betekent **1** dat u het **helemaal oneens** bent en **9** dat u het **helemaal eens** bent met het opnemen van deze ‘**HOE**’-boodschap. Daarnaast kunt u in het opmerkingenveld de keuze voor uw antwoord toelichten en de formulering van deze boodschap zonodig herschrijven.

NB: de pedagogische boodschappen zijn in willekeurige volgorde opgesteld. Dit betekent dat een boodschap die op nummer 1 staat niet belangrijker of juist minder belangrijk is dan een boodschap die op 10 staat.

| **Top 10 van pedagogische boodschappen** | **Deze ‘HOE’- boodschap ter preventie van overgewicht bij kinderen 4-13 moet opgenomen worden in de pedagogische boodschap.**  **Noteer hieronder een cijfer tussen de 1-9 .** | **Opmerkingen: licht uw antwoord toe.** |
| --- | --- | --- |
| ‘**WAT**’-boodschap:   1. Een competente/authoritatieve opvoedingsstijl is erg belangrijk. |  |  |
| ‘**HOE**’-boodschappen: |  |  |
| - Zorg zoveel mogelijk voor structuur binnen het gezin, bijvoorbeeld door eetmomenten gezamenlijk (aan tafel) door te brengen of door afspraken te maken over wanneer er bijvoorbeeld tv gekeken mag worden. |  |  |
| - Praat met uw kind over het belang van gezond eten en voldoende beweging, zonder de nadruk te leggen op afvallen. |  |  |
| - Maak afspraken met je kind over gezond eten (bijvoorbeeld hoeveel happen proeven) en tv kijken, computeren en bewegen. Maak de gemaakte afspraken ook zichtbaar, door ze bijvoorbeeld op te hangen aan de muur. |  |  |
| - Als u uw kind duidelijk heeft gemaakt wat u van uw kind verlangt en uw kind weet dat de afspraken zullen worden nageleefd, is het voor uw kind makkelijker zich aan de afspraken te houden. Beloon dan ook uw kind wanneer het zich aan de afspraken houdt door bijvoorbeeld samen iets leuks te gaan doen. |  |  |
| ‘**WAT**’-boodschap:   1. Als ouder dient u het goede voorbeeld te geven. |  |  |
| ‘**HOE**’-boodschappen: |  |  |
| - Uitleg: Kinderen zijn snel geneigd het gedrag van hun ouders te imiteren. Als u laat zien dat gezonde voeding lekker is en bewegen leuk is, zal uw kind dit ook volgen. |  |  |
| - Ga dus zelf niet televisie kijken, terwijl u net tegen uw kind heeft gezegd dat hij/zij moet gaan bewegen. |  |  |
| - Hetzelfde geldt voor voeding: uw kind zal lastig accepteren dat hij/zij geen frisdrank mag drinken, terwijl u het wel doet waar hij/zij bij is. |  |  |
| - Zorg in ieder geval voor dat ongezonde voeding (bijvoorbeeld snoep/chips) minder zichtbaar is, dan gezondere voeding, zoals fruit. Zet bijvoorbeeld een gevulde fruitschaal op tafel en verberg de snoeptrommel. |  |  |
| - Dit geldt ook voor bewegingsactiviteiten: zorg dat bijvoorbeeld apparaten als de iPad minder zichtbaar zijn, terwijl u bewegingsactiviteiten zoals een fiets of bal meer zichtbaar maakt. |  |  |
| ‘**WAT**’-boodschap:   1. Minstens 60 minuten per dag bewegen is erg belangrijk voor uw kind. |  |  |
| ‘**HOE**’-boodschappen: |  |  |
| - Maak het bewegen leuk door zelf mee te doen of door vriendjes of broertjes/zusjes te vragen mee te doen. |  |  |
| - Bewegen kan op veel verschillende manieren, zoals een boswandeling, een balspel, zwemmen of door uw kind buiten te laten spelen. |  |  |
| - Probeer tevens, wanneer dit mogelijk is, de auto zoveel mogelijk te laten staan. Wanneer uw kind fietst of loopt, bijvoorbeeld naar de supermarkt of naar school, krijgt hij/zij ook meer beweging. |  |  |
| ‘**WAT**’-boodschap:   1. Naast voldoende beweging per dag, is minstens 1x per week sporten ook erg belangrijk voor uw kind. |  |  |
| ‘**HOE**’-boodschappen: |  |  |
| - Uitleg: Niet alleen krijgt uw kind op deze manier veel beweging, maar ook komt uw kind in contact met andere kinderen en leert hij/zij kind discipline en omgang met andere kinderen. |  |  |
| - Ga uw kind niet dwingen om te sporten, maar laat wel zien dat het leuk is. |  |  |
| - Overleg met uw kind welke sport hij/zij leuk vindt en laat uw kind, als dit nodig is, diverse sportlessen volgen, zodat uw kind erachter kan komen wat hij/zij leuk vindt. |  |  |
| - Indien u niet over voldoende financiële mogelijkheden beschikt om uw kind op een sport te doen, kunt u bij uw gemeente informeren naar financiële ondersteuning. |  |  |
| - NB: ook wanneer uw kind 1x per week sport, blijft beweging iedere dag belangrijk! |  |  |
| ‘**WAT**’-boodschap:   1. Ontbijt iedere dag. |  |  |
| ‘**HOE**’-boodschappen: |  |  |
| - Uitleg: Door te ontbijten komt de stofwisseling van uw kind goed op gang en daarnaast kan het zich beter concentreren op school en zal het minder snel trek krijgen. |  |  |
| - Geef uw kind het goede voorbeeld door zelf ook dagelijks te ontbijten. |  |  |
| - Naast ontbijten, is het ook belangrijk dat er wordt geluncht en dat er gezond avondeten is. Hierdoor raakt uw kind gewend aan structuur en heeft hij/zij minder behoefte aan eetmomenten tussendoor. Drie tussendoortjes gedurende de dag zijn dan voldoende. |  |  |
| ‘**WAT**’-boodschap:   1. Probeer te variëren met voeding, zodat het kind voldoende voedingsstoffen binnenkrijgt. |  |  |
| ‘**HOE**’-boodschappen: |  |  |
| - Varieer zelf ook met eten. Wanneer u een sterke voorkeur heeft voor bepaalde producten, zal uw kind niet inzien waarom het zelf gevarieerd moet eten. |  |  |
| - U kunt variatie aanbieden door uw kind verschillende soorten fruit, groente en maaltijden te laten proeven. |  |  |
| - Maak het proeven leuk door bijvoorbeeld uw kind mee te laten helpen met koken. |  |  |
| ‘**WAT**’-boodschap:   1. Beperk het aantal glazen frisdrank. |  |  |
| ‘**HOE**’-boodschappen: |  |  |
| - Uitleg: In frisdranken zoals cola, maar ook in vruchtensappen (appelsap, Multi-vitamine drank), ranja en yoghurtdranken, zitten veel suikers die slecht zijn voor de gezondheid van uw kind. Ze werken niet alleen overgewicht in de hand, maar zijn ook erg slecht voor het gebit. |  |  |
| - Haal deze zoete dranken dan ook zo min mogelijk in huis, zodat uw kind ook niet in de verleiding wordt gebracht. Geef hierin ook zelf het goede voorbeeld door zelf ook suikervrije of minder zoete drankjes te drinken, zoals water, thee zonder suiker, zoete dranken aangelengd met veel water. |  |  |
| - Maak afspraken over wanneer uw kind frisdrank mag drinken. Een keer per dag is voldoende, dus bijvoorbeeld na schooltijd of ’s avonds in het weekend. |  |  |
| - Kijk ook uit met light producten: hoewel deze producten vaak minder suiker bevatten, worden andere zoetstoffen toegevoegd die ook schadelijk kunnen zijn. Daarnaast zitten ook in light dranken vetten en bevatten ze niet altijd 0 calorieën. |  |  |
| - Laat uw kind naast 2 bekers melk, water, thee (liefst zonder suiker, maar het kan wel met een smaakje) of vruchtensap aangelengd met veel water drinken. |  |  |

| ‘**WAT**’-boodschap:   1. Beperk het aantal snacks en tussendoortjes. |  |  |
| --- | --- | --- |
| ‘**HOE**’-boodschappen: |  |  |
| - Uitleg: In chips, snoep maar ook in koekjes zitten veel suikers en vetten, welke slecht zijn voor uw kind. Ze werken niet alleen overgewicht in de hand, maar zijn ook erg slecht voor het gebit. |  |  |
| - Ook koekjes *speciaal voor kinderen’* bevatten veel zoetstoffen en vetten die slecht zijn voor uw kind. Daarnaast zijn kinderkoeken vaak per twee of drie verpakt, terwijl één zo’n koekje al genoeg is voor uw kind. Houdt hier dus rekening mee. |  |  |
| - Geef uw kind fruit, een rijstewafel, tomaatjes of stukjes komkommer als tussendoortje. |  |  |
| - Bouw een vast moment in de week in waarop uw kind iets lekkers mag, zoals een bakje chips. Hierdoor raakt uw kind gewend aan de regelmaat. |  |  |
| - Probeer verder maximaal drie (gezonde) tussendoortjes per dag te geven. |  |  |
| ‘**WAT**’-boodschap:   1. Laat uw kind niet langer dan twee uur per dag tv kijken, computeren of op apparaten als de iPad of Xbox. |  |  |
| ‘**HOE**’-boodschappen: |  |  |
| - Uitleg: Door deze zittende activiteiten beweegt uw kind niet en verbrandt weinig calorieën. |  |  |
| - Uitleg: Daarnaast gaat bijvoorbeeld tv kijken vaak gepaard met (onbewust) eten van snacks. |  |  |
| - Maak afspraken met uw kind over wanneer het tv mag kijken of mag computeren en maak deze afspraken ook zichtbaar. Hang de afspraken bijvoorbeeld op de koelkast of naast de tv. |  |  |
| - Biedt ook alternatieve activiteiten aan uw kind aan (beweegspelletjes) voor wanneer uw kind geen tv mag kijken. |  |  |
| ‘**WAT**’-boodschap:   1. Geen tv of apparaten als de iPad tijdens de maaltijden. |  |  |
| ‘**HOE**’-boodschappen: |  |  |
| - Maak aan uw kind duidelijk dat er geen tv aan mag tijdens het ontbijt of ander eetmoment en hou uzelf daar ook aan. |  |  |
| - Uitleg: Als de tv aan staat tijdens de maaltijd, wordt er onbewust gegeten en is de kans groot dat uw kind meer eet dan eigenlijk nodig is. |  |  |

1. Welke pedagogische boodschap(pen) mist u nog?

| **Andere pedagogische boodschappen** | **Licht uw antwoord toe** |
| --- | --- |
|  |  |
|  |  |
|  |  |
|  |  |
|  |  |

**Dit is het einde van de 2^e^ vragenlijst.**

**Hartelijk bedankt voor het invullen van deze vragenlijst. Zou u de ingevulde vragenlijst vóór *<datum>* naar ons willen retourneren via:**

**Mocht u vragen en/of opmerkingen hebben dan kunt u deze hieronder kwijt.**

|  |
| --- |

Voor vragen over de vragenlijst en/of het invullen daarvan kunt u contact opnemen met:
