# Supplementary material for: A set of pedagogical recommendations for improving the integrated approach to childhood overweight and obesity: A Delphi study
Source: PLoS One. 2020 Apr 27;15(4):e0231245. doi: 10.1371/journal.pone.0231245 (PMC7185684; doi:10.1371/journal.pone.0231245)
Supplement: S3 File — (DOC) [file pone.0231245.s003.doc]

| 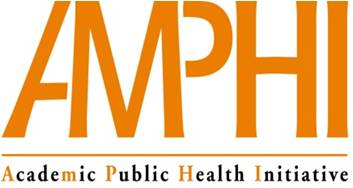 |  |
| --- | --- |

**Questionnaire round 2**

**June 2013**

**Gain consensus on the content of a pedagogic message for parents to prevent overweight in children between 4 and 13 years old: a Delphi study**

**Introduction**

The results of the first round of our Delphi study show that there is already a lot of consensus among the various participating professionals in our Delphi study. Below you will find the combined measures that you all agree are important in a pedagogical message to parents:

1. Parenting
   1. Stimulating a competent parenting style, or an authoritative parenting style. *What you all find fit under a competent parenting style is*:

i. Structure (within the household and family);

ii. Clear rules and agreements and be consistent;

iii. Giving love, warmth and attention to your child, doing fun things together;

iv. Listening to your child;

v. Rewarding the child or giving compliments when he / she does something good.

- 1. Be a good role model as a parent.
  2. Food should not be used as a sweetener to comfort, distract or reward your child.
  3. Do not emphasize weight loss / dieting.
  4. Ensure that unhealthy products or activities (such as chips, the iPad) are not visible, while healthy products and activities (such as fruit, a bicycle or ball) are visible.

*Promote healthy eating by the points mentioned under parenting.*

1. Diet
   1. Breakfast every day, preferably together and at the table.
   2. 3 main meals and a maximum of 3 snacks at set times, preferably together at the table. For the quantities, see the guidelines of the nutrition center.
   3. Vary with food/your diet so that your child gets enough different nutrients. It is also important that you let your child taste everything.
2. Soft drinks and candy/snacks
   1. Limit the intake of sweet drinks such as fruit juices, ranja, ice tea and soft drinks. *There is still no consensus on a specific drinking recommendation. This can be per day: 2 glasses of milk, 1 glass of sweet (eg ranja / fruit juice / multivitamin / soft drink) other water, tea without sugar. Preferably mix fruit juices with water.*
   2. Limit sweets / snacks. *There is still no consensus about how often snacks / snacks.*

*Promote adequate exercise and reduce inactivity by the points mentioned under parenting.*

1. Physical activity
   1. 60 minutes of physical activity a day at least: such as walking (to school) or cycling, running, swimming, walking the dog.
   2. Exercising together with the family or with friends, it have to be fun.
   3. Decide together with your child which form of exercise he/she likes.
   4. Ask the municipality for financial assistance for sports grants, when you need it.
2. Tv and computer
   1. Watch TV and use the computer for no more than 2 hours a day. This also applies to devices such as the Xbox and the iPad.
   2. No TV in the children's bedroom.

In addition, you also had comments and/or additions to the advice given to parents from the "SO that BOFT factors" and the guidelines from the "NHG patient letter". However, there were also opinions that you all felt were less important.

This second questionnaire was drawn up on the basis of the above findings.

**Thank you in advance for your answers!**

**Questions:**

1. The following advice was considered by most participating professionals as NOT important or NOT effective enough to be included in a pedagogical message to prevent childhood obesity. Can you indicate per advice to what extent you agree that we will **NOT** include this advice in the pedagogical message? You can indicate this on a **scale from 1 to 9**. Here **1** means that you **completely disagree** and **9** that you completely agree with not taking this advice. You can also explain the choice of your answer in the comments field.

| **Advice to parents** | **This advice for the prevention of overweight in children 4-13 should NOT be included in the pedagogical message.**  **Write a number between 1-9 below.** | **Comments: explain your answer.** |
| --- | --- | --- |
| Encouraging adequate and regular sleep |  |  |
| Encourage at least 10 hours of sleep per night |  |  |
| Make your child a member of a sports club and regularly watch training sessions or competitions to stimulate it. |  |  |
| If your child has achieved swimming diploma A, have your child pass for diploma B and C. |  |  |
| Vitamin pills are not required. |  |  |
| Only allow your child to eat sweets at parties or at the weekend, and then only a little. |  |  |

1. *Below is a top 10 of possible pedagogical messages that professionals can spread to parents so that they know how to prevent overweight in children between 4 and 13 years old. These messages are constructed from the advice on which consensus has been reached. The messages are subdivided into a "****WHAT****" message and a "****HOW****" message. The "****WHAT****" message consists of concrete messages, which have been compiled on the basis of aggregated opinions on which consensus has been reached. In the "****HOW****" message pedagogical tips, based on the opinions on which you have reached consensus, are given to explain the "WHAT" message to parents.*

Can you indicate per "**HOW**" message to what extent you agree that we will include this advice in the pedagogical message? You can indicate this on a **scale from 1 to 9**. Here **1** means that you **completely disagree** and **9** that you **completely agree** with the recording of this "**HOW**" message. In addition, you can explain the choice for your answer in the comments field and, if necessary, rewrite the wording of this message.

Note: the pedagogical messages are arranged in random order. This means that a message that is number 1 is no more important or less important than a message that is number 10.

| **Top 10 pedagogical messages** | **This "HOW" message for the prevention of overweight in children 4-13 must be included in the pedagogical message.**  **Write a number between 1-9 below.** | **Comments: explain your answer.** |
| --- | --- | --- |
| "**WHAT**" message:   1. A competent / authoritative upbringing style is very important. |  |  |
| "**HOW**" messages: |  |  |
| - Provide as much structure as possible within the family, for example by spending eating moments together (at the table) or by making agreements about when, for example, at what moments the TV can be watched. |  |  |
| - Talk to your child about the importance of healthy eating and adequate exercise, without emphasizing weight loss. |  |  |
| - Make agreements with your child about healthy diet (for example how many bites taste) and watching TV, using the computer and exercising. Make these agreements visible, for example, by hanging them on the wall. |  |  |
| - If you have made it clear to your child what you expect from your child and your child knows that the agreements will be complied with, it is easier for your child to adhere to the agreements. Reward your child when he / she keeps to the agreements, for example by doing something fun together. |  |  |
| "**WHAT**" message:   1. As a parent you must be a good role example. |  |  |
| "**HOW**" messages: |  |  |
| - Explanation: Children tend to imitate the behavior of their parents. If you show that healthy diet is tasty and exercise is fun, your child will also follow this. |  |  |
| - Do not watch television yourself, if you just told your child to be physical active. |  |  |
| - The same applies to diet: it is difficult for your child to accept that he / she is not allowed to drink soft drinks, while you do it while he / she is present. |  |  |
| - Make sure that unhealthy diet (such as candy / chips) is less visible than healthier diet, such as fruit. For example, put a filled fruit bowl on the table and hide the candy box. |  |  |
| - This also applies to physical activity/exercise: make devices such as the iPad less visible, while you make physical activity instruments, such as a bicycle or a ball more visible. |  |  |
| "**WHAT**" message:   1. Being physical active for at least 60 minutes a day is very important for your child. |  |  |
| "**HOW**" messages: |  |  |
| - Make being physical active/ the exercise fun by participating yourself or by asking friends or brothers / sisters to participate. |  |  |
| - Being physical active can be done in many different ways, such as a walk in the forest, a ball game, swimming or by having your child play outside. |  |  |
| - Whenever possible, also try to leave the car as far as possible. When your child cycles or walks, for example to the supermarket or to school, he / she also gets more exercise. |  |  |
| "**WHAT**" message:   1. In addition to being sufficient physical active per day, exercising (playing sports) at least once a week is also very important for your child. |  |  |
| "**HOW**" messages: |  |  |
| - Explanation: Not only does your child get a lot of exercise this way, but your child also comes into contact with other children and he / she learns child discipline and how to interact with other children. |  |  |
| - Do not force your child to play sports, but do show that it is fun. |  |  |
| - Discuss with your child which sport he / she likes and have your child follow various sports lessons if necessary so that your child can find out what he / she likes. |  |  |
| - If you do not have sufficient financial options to put your child in a sport, you can ask your municipality for financial support. |  |  |
| - NB: even when your child does exercise/play sport once a week, being physical active remains important every day! |  |  |
| "**WHAT**" message:   1. Eat breakfast daily. |  |  |
| "**HOW**" messages: |  |  |
| - Explanation: Your child's metabolism will get off to a good start with breakfast, and in addition it will be better able to concentrate at school and less likely to get hungry. |  |  |
| - Be a good role model by giving your child a daily breakfast. |  |  |
| - In addition to breakfast, it is also important to have lunch and to have a healthy dinner. This way your child gets used to structure and he / she has less need for eating moments in between. Three snacks during the day are then sufficient. |  |  |
| "**WHAT**" message:   1. Try to vary with food products so that the child gets enough nutrients. |  |  |
| "**HOW**" messages: |  |  |
| - Also vary with food products. If you have a strong preference for certain products, your child will not see why he or she must eat a varied diet. |  |  |
| - You can offer variation by having your child taste different types of fruit, vegetables and meals. |  |  |
| - Make the tasting fun by, for example, letting your child help cook. |  |  |
| "**WHAT**" message:   1. Limit the number of glasses of sugar-sweetened drinks. |  |  |
| "**HOW**" messages: |  |  |
| - Explanation: In sugar-sweetend drinks such as cola, but also in fruit juices (apple juice, multi-vitamin drink), ranja and yogurt drinks, there are many sugars that are bad for your child's health. They not only promote obesity, but are also very bad for the teeth. |  |  |
| - Get these sugar-sweetened drinks as little as possible at home, so that your child will not be tempted. Be a good role model yourself by also drinking sugar-free or less sugar-sweetened drinks, such as water, tea without sugar, sugar-sweetened drinks diluted with plenty of water. |  |  |
| - Make agreements about when your child can drink soft drinks. Once a day is sufficient, for example after school or in the evening during the weekend. |  |  |
| - Also be careful with light products: although these products often contain less sugar, other sweeteners are added that can also be harmful. In addition, light drinks can also contain fats and do not always contain 0 calories. |  |  |
| - Have your child in addition to 2 cups of milk, drink water, tea (preferably without sugar, but with a taste) or fruit juice diluted with plenty of water. |  |  |

| "**WHAT**" message:   1. Limit the number of snacks. |  |  |
| --- | --- | --- |
| "**HOW**" messages: |  |  |
| - Explanation: There are many sugars and fats in chips, candy and cookies, which are bad for your child. They not only promote obesity, but are also very bad for the teeth. |  |  |
| - Cookies “*specially for children*" also contain many sweeteners and fats that are bad for your child. In addition, children's cookies are often packaged in pairs or three, while one is enough for your child. So keep this in mind. |  |  |
| - Give your child fruit, a rice cake, tomatoes or pieces of cucumber as a snack. |  |  |
| - Select a fixed time in the week when your child can eat a cookie of snacks, such as a bowl of chips. This way your child gets used to the regularity. |  |  |
| - Furthermore, try to give a maximum of three (healthy) snacks per day. |  |  |
| "**WHAT**" message:   1. Do not allow your child to watch TV, use the computer or on devices such as the iPad or Xbox for more than two hours a day. |  |  |
| "**HOW**" messages: |  |  |
| - Explanation: During these sedentary activities, your child does not move and does not burn a lot of calories. |  |  |
| - Explanation: In addition, watching TV is often accompanied by (unconsciously) eating snacks. |  |  |
| - Make agreements with your child about when they can watch TV or use computers and make these agreements visible. For example, hang the appointments on the refrigerator or next to the TV. |  |  |
| - Also offer alternative activities to your child (exercise games) for when your child is not allowed to watch TV. |  |  |
| "**WHAT**" message:   1. No TV or devices like the iPad during meals. |  |  |
| "**HOW**" messages: |  |  |
| - Make it clear to your child that it is not allowed to watch TV during breakfast/lunch/dinner and stick to it yourself. |  |  |
| - Explanation: If the TV is on during the meals, people eat unknowingly/unconsciously and there is a good chance that your child will eat more than is actually necessary. |  |  |

1. Which pedagogical message (s) do you still miss?

| **Other general parenting rules** | **Please explain your answer** |
| --- | --- |
|  |  |
|  |  |
|  |  |
|  |  |
|  |  |

**This is the end of the 1st questionnaire.**

**Thank you for completing this questionnaire. Would you like to return the completed questionnaire to us before <date> via:**

**If you have any questions and / or comments, you can enter them below.**

|  |
| --- |

For questions about the questionnaire and / or how to complete it, please contact:
