# Supplementary material for: A set of pedagogical recommendations for improving the integrated approach to childhood overweight and obesity: A Delphi study
Source: PLoS One. 2020 Apr 27;15(4):e0231245. doi: 10.1371/journal.pone.0231245 (PMC7185684; doi:10.1371/journal.pone.0231245)
Supplement: S4 File — (DOC) [file pone.0231245.s004.doc]

| 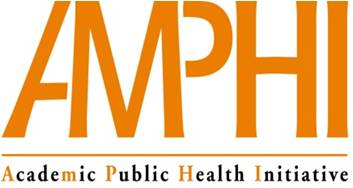 |  |
| --- | --- |

**Questionnaire round 1**

**March 2013**

**Gain consensus on the content of a pedagogic message for parents to prevent overweight in children between 4 and 13 years old: a Delphi study**

**Introduction**

From the literature and guidelines regarding (the prevention of) overweight in children, a number of important determinants and accompanying recommendations emerge that we will list below for you. For example, the Youth Health Care (YHC), the Nutrition Center and the Netherlands Institute for Sport and Exercise (NISB) use the **SO** that **(B) BOFT** factors. In addition, the Dutch Society for General Practitioners (NHG) has drawn up a number of guidelines for the prevention of overweight in children, the NHG patient letter: "Overweight in children". In addition to the organizations mentioned above, there are more professionals, institutions and stakeholders who have drawn up rules and guidelines or formulated opinions for the prevention of overweight in children between 4 and 13 years old.

| The abbreviation ‘**SO** that **(B)BOFT**’ stands for: | Related **advice to parents** are**:** |
| --- | --- |
| - Encouraging adequate and regular **S**leep | - At least 10 hours of sleep per night |
| - Stimulate competent parenting styles (in Dutch = **O**pvoeding) | - Clear rules and agreements with your child and at the same time give love and warmth. |
| - Promoting being physical active and playing outside daily (in Dutch = **B**uitenspelen) | - Parents be physical active yourself, so that children have an good example. - Less car use. - > 1 hour / day of cycling, walking or exercising. - Swimming, cycling or walking with children. |
| - Eat breakfast daily (in Dutch = **O**ntbijt) | - Breakfast preferably in a family context. - Offer a healthy breakfast. - No TV watching during breakfast. |
| - **F**resh water from the tap, leave soft drinks and other sugar-sweetened drinks | - Do not buy sugar-sweetened drinks. - No or a maximum of 1 glass of sugar-sweetened soft drinks per day. - Children drink water, tea or fruit juice mixed with water. |
| - **T**v and computer? Say more often: NO. nee. | - Children do not watch TV and / or computers for more than 2 hours a day. - Never watch TV or use a computer for longer than being physical active. - No TV in the children's bedroom. |

Stimulating **B**reastfeeding also belongs to these rules, but because we want to focus on the age group between 4 and 13, this rule is not taken into consideration.

The determinants in the NHG patient letter largely correspond to the **SO** that **BOFT** factors used by the JGZ, the nutrition center and the NISB.

| The determinants that the NHG sets in the **NHG patient letter: Overweight in children** are: | Related **advice to parents** are: |
| --- | --- |
| - More physical activity for an overweight child | - Make arrangements with your child to ensure that he or she does not sit in front of the computer- or TV-screen for too long. - Make sure your child plays outside regularly, goes shopping on a bicycle or walks the dog. - A child must be physical active for at least an hour every day. Don't force it but join in and make it fun. Also ask friends, brothers or sisters to participate. - Make your child a member of a sports club and regularly watch training sessions or competitions to stimulate it. - If your child has achieved swimming diploma A, have your child pass for diploma B and C. - Undertake physical activities that you can do with the whole family, such as a forest walk, a beach walk or a bike ride together or swimming together at the weekend. - Walk to school together, take the bicycle more often instead of the tram, bus or car. - Every little bit of being physical active helps. The foods that your child receives are then consumed better. Your child gets more and more muscle and less fat. It gets out of breath less quickly and feels fitter. |
| - Healthy diet for an overweight child | To ensure that your child does not become overweight, it is necessary that it starts eating differently.   - Ensure variety in diet. Vegetable, fruit and whole grain products contain many valuable nutrients and few calories. They give a feeling of fullness, which means your child is less likely to eat too much. - Choose lean meat and low-fat or semi-skimmed milk products. - Use low fat when cooking. - Drinking about one liter a day is important: in addition to milk, especially water or tea without sugar, and sometimes also fresh juice. - By eating a healthy and varied diet your child will get all the nutrients it needs. - Vitamin pills are not required. - You can enter the age and gender of your child on the Nutrition Center website. Then you will get an overview of what your child can eat best each day. - Eat at set times. - Do not skip breakfast. - Three meals a day is fine. - Give a healthy snack at most four times a day, for example: fruit, wholemeal biscuit, breadstick or rice cake, a box of raisins, pieces of cucumber or carrot. Give as little soft drinks, candy or chips as possible. - Only allow your child to eat sweets at parties or at the weekend, and then only a little. - Give the right example yourself. Participate in regularity and healthy diet. - Do not use food as a sweetener to distract, comfort or reward your child. - Give your child attention, listen to what it has to say, give it an extra hug or read a book together. If a child gets the right attention, it has less need to eat. |

**Questions:**

*First we ask you something about your familiarity with and your opinion about the content of the above-mentioned SO that BOFT factors and the NHG patient letter: "Overweight in children".*

1. Are you familiar with the SO that BOFT factors? Choose yes or no. In addition, you can explain the choice for your answer in the comments field if necessary.

| **Answer** | **Comments** |
| --- | --- |
| Yes/No  *(Delete the answer that does not apply).* | *Enter your comments here.* |

1. For each SO that BOFT factor can you indicate to what extent you agree with the effectiveness of this factor for the prevention of overweight in children? You can indicate this on a **scale from 1 to 9**. Here **1** means that you **completely disagree** and **9** that you **completely agree** with the effectiveness of this factor. You can also explain the choice of your answer in the comments field.

| **SO that BOFT factors** | **This factor for the prevention of overweight in children 4-13 years is very effective.**  **Write a number between 1-9 below.** | **Comments: explain your answer.** |
| --- | --- | --- |
| Encouraging adequate and regular sleep |  |  |
| Stimulate competent parenting styles |  |  |
| Promoting being physical active and playing outside daily |  |  |
| Eat breakfast daily |  |  |
| Fresh water from the tap, leave soft drinks and other sugar-sweetened drinks |  |  |
| Tv and computer? Say more often: NO. nee. |  |  |

1. Are you familiar with the NHG patient letter: "Overweight in children"? Choose yes or no. In addition, you can explain the choice for your answer in the comments field if necessary.

| **Answer** | **Comments** |
| --- | --- |
| Yes/No  (Delete the answer that does not apply). | *Enter your comments here.* |

1. For the advice to parents associated with the **"SO that BOFT factors"** and the **"NHG patient letter:" Overweight in children "**, can you indicate to what extent you think these recommendations are effective in preventing overweight in children? You can indicate this on a **scale from 1 to 9**. Here *1* means that you **completely disagree** and **9** that you **completely agree** with the effectiveness of this factor. You can also explain the choice of your answer in the comments field.

| **Advice to parents** | **This factor for the prevention of overweight in children 4-13 years is very effective.**  **Write a number between 1-9 below.** | **Comments: explain your answer.** |
| --- | --- | --- |
| Encourage at least 10 hours of sleep per night |  |  |
| Clear rules and agreements with your child and at the same time give love and warmth. |  |  |
| Parents be physical active yourself, so that children have an good example. |  |  |
| Less car use. |  |  |
| > 1 hour / day of cycling, walking or exercising. |  |  |
| Swimming, cycling or walking with children. |  |  |
| Breakfast preferably in a family context. |  |  |
| Offer a healthy breakfast. |  |  |
| No TV watching during breakfast. |  |  |
| Do not buy sugar-sweetened drinks. |  |  |
| No or a maximum of 1 glass of sugar-sweetened soft drinks per day. |  |  |
| Children drink water, tea or fruit juice mixed with water. |  |  |
| Children do not watch TV and / or computers for more than 2 hours a day. |  |  |
| Never watch TV or use a computer for longer than being physical active. |  |  |
| No TV in the children's bedroom. |  |  |
| Make arrangements with your child to ensure that he or she does not sit in front of the computer- or TV-screen for too long. |  |  |
| Make sure your child plays outside regularly, goes shopping on a bicycle or walks the dog. |  |  |
| A child must be physical active for at least an hour every day. Don't force it but join in and make it fun. Also ask friends, brothers or sisters to participate. |  |  |
| Make your child a member of a sports club and regularly watch training sessions or competitions to stimulate it. |  |  |
| If your child has achieved swimming diploma A, have your child pass for diploma B and C. |  |  |
| Undertake physical activities that you can do with the whole family, such as a forest walk, a beach walk or a bike ride together or swimming together at the weekend. |  |  |
| Walk to school together, take the bicycle more often instead of the tram, bus or car. |  |  |
| Every little bit of being physical active helps. The foods that your child receives are then consumed better. Your child gets more and more muscle and less fat. It gets out of breath less quickly and feels fitter. |  |  |
| Ensure variety in diet. Vegetable, fruit and whole grain products contain many valuable nutrients and few calories. They give a feeling of fullness, which means your child is less likely to eat too much. |  |  |
| Choose lean meat and low-fat or semi-skimmed milk products. |  |  |
| Use low fat when cooking. |  |  |
| Drinking about one liter a day is important: in addition to milk, especially water or tea without sugar, and sometimes also fresh juice. |  |  |
| By eating a healthy and varied diet your child will get all the nutrients it needs. |  |  |
| Vitamin pills are not required. |  |  |
| You can enter the age and gender of your child on the Nutrition Center website. Then you will get an overview of what your child can eat best each day. |  |  |
| Eat at set times. |  |  |
| Do not skip breakfast. |  |  |
| Three meals a day is fine.  Give a healthy snack at most four times a day, for example: fruit, wholemeal biscuit, breadstick or rice cake, a box of raisins, pieces of cucumber or carrot. Give as little soft drinks, candy or chips as possible. |  |  |
| Only allow your child to eat sweets at parties or at the weekend, and then only a little. |  |  |
| Give the right example yourself. Participate in a regularity and healthy diet. |  |  |
| Do not use food as a sweetener to distract, comfort or reward your child. |  |  |
| Give your child attention, listen to what it has to say, give it an extra hug or read a book together. If a child gets the right attention, it has less need to eat. |  |  |

1. As already mentioned in the introduction, as an expert you can also have advice and guidelines in your own professional group, or you can have your own ideas about the pedagogical message to parents to prevent being overweight in children between 4 and 13 years old. These ideas need neither scientific basis nor support from other experts. What advice, from other organizations or yourself, do you still miss and would you like to add? Please explain your answer.

| **Other advice or your own ideas** | **Please explain your answer** |
| --- | --- |
|  |  |
|  |  |
|  |  |
|  |  |
|  |  |
|  |  |
|  |  |
|  |  |
|  |  |

1. If you look at the aforementioned factors, guidelines and advice to parents, are there (still) general pedagogical advice that helps parents in the daily situation to get their child to eat healthier and to exercise sufficiently? This therefore concerns general upbringing (general parenting): the extent to which parents make clear agreements with their child, set rules, support, reward and punish.

| **Other general parenting rules** | **Please explain your answer** |
| --- | --- |
|  |  |
|  |  |
|  |  |
|  |  |
|  |  |
|  |  |
|  |  |
|  |  |
|  |  |
|  |  |
|  |  |
|  |  |
|  |  |

1. Can you make a top 5 of the advice / messages to parents to prevent overweight in children between 4 and 13 years old, which you think are the most important? Start with the advice / message that you find most important.

| **Top 5 advice / messages to parents for the prevention of overweight in children between the ages of 4 and 13** | **Please explain your answer** |
| --- | --- |
| 1. |  |
| 2. |  |
| 3. |  |
| 4. |  |
| 5. |  |

1. Do you know any experts that you think we should approach for this or the next round of this Delphi study? Would you note the name, position, e-mail address and possibly telephone number of this person, so that we can invite the person in question to participate in our research.

Full name:

Function / profession:

E-mail address:

Telephone number:

**This is the end of the 1st questionnaire.**

**Thank you for completing this questionnaire. Would you like to return the completed questionnaire to us before <date> via:**

If you have any questions and / or comments, you can enter them below.

|  |
| --- |

For questions about the questionnaire and / or how to complete it, please contact:
